# The application of novel techniques in ophthalmology education

**DOI:** 10.3389/fmed.2024.1459097

**Published:** 2024-11-14

**Authors:** Yang Jiang, Hanyu Jiang, Zhikun Yang, Ying Li, Youxin Chen

**Affiliations:** ^1^Department of Ophthalmology, Peking Union Medical College Hospital, Chinese Academy of Medical Sciences, Beijing, China; ^2^Key Laboratory of Ocular Fundus Diseases, Chinese Academy of Medical Sciences & Peking Union Medical College, Beijing, China; ^3^Eight-year Medical Doctor Program, Chinese Academy of Medical Sciences & Peking Union Medical College, Beijing, China

**Keywords:** three-dimensional, artificial intelligence, virtual reality, ophthalmology, education

## Abstract

This paper synthesizes recent advances of technologies in ophthalmology education. Advancements in three-dimensional technology are revolutionizing ophthalmology education by enhancing the visualization, understanding, and retention of complex anatomical and pathological concepts. In addition to physical models, artificial intelligence and virtual reality are emerging as significant tools. A systematic search of PubMed was carried out, with a search date from inception to 01/05/2024. A total of 6,686 articles were screened, of which 6,470 were excluded following abstract review. After reading the remaining 216 articles in full, a further 186 were excluded. A total of 30 original articles were included in the review. This review underscores the transformative impact of novel technology in ophthalmology education, offering innovative solutions to enhance learning, surgical training, and diagnostic skills. Further research and development in this field hold promise for continued improvements in ophthalmology education and practice.

## 1 Introduction

Vision is one of the most important senses. Blindness is ranked by the public to be the worst disease (1). Accurate diagnosis and effective treatment can improve the quality of life by preserving or enhancing vision, allowing individuals to continue performing daily tasks. Ophthalmology is a complex and specialized field, which requires in-depth knowledge of the anatomy, physiology, and diseases of the eye. Ophthalmology education is essential for ensuring that healthcare professionals have the knowledge, skills, and expertise needed to provide high-quality care to patients with eye conditions.

However, there are some challenges in ophthalmology education. First, an imbalance of educational resources in ophthalmology results in disparities in access to quality education, training, and resources in the field. Rural and underserved areas often lack access to specialized ophthalmic training programs. Limited financial resources can affect the ability of junior doctors to pursue education and training in ophthalmology, particularly in low-income countries where there may be insufficient funding for educational programs. Unequal distribution of modern equipment, technology, and facilities can affect the quality of ophthalmology education and training, with some institutions having superior resources to others. Second, ophthalmology training programs include a combination of theoretical study and clinical practice. Balancing these demands can be challenging for trainees, as they may struggle to find time. Finally, the education and training of ophthalmologists may vary in terms of curriculum, quality, and duration across institutions and countries. This lack of standardization can result in inconsistencies in the level of knowledge and skills among ophthalmologists, which affects the quality of patient care.

Recently, new techniques are emerging in medical education. These advances have a profound influence on medical education, transforming the way students and trainees learn and practice medicine. Advances in ophthalmology education have the potential to solve current challenges in ophthalmology education. This review has been conducted to evaluate these novel technologies.

## 2 Methods

### 2.1 Eligibility criteria

Original studies were included if they described developments in ophthalmic training and met the following criteria: (1) study participants were ophthalmologists or medical students in ophthalmology and (2) educational studies. Studies were excluded if: (1) they did not include original data; (2) the studies were not specific to ophthalmology; or (3) the studies were not written in English.

### 2.2 Search methods

A systematic search of PubMed was carried out, using the terms “(train* OR education) AND ophthalm*.” The search date was from inception to 01/05/2024. Reference lists from included articles and relevant reviews were searched for eligible studies.

### 2.3 Study selection

Two authors carried out independent, duplicate searches. All abstracts were reviewed, and articles that were potentially eligible were read in full. The final list of studies that met the eligibility criteria was compared, and any disagreements were resolved through discussion.

### 2.4 Data collection

The same two authors extracted data for each study separately, and differences were resolved through discussion. Data collected included details of the training objective, participants, assessment, and advantages and disadvantages.

## 3 Results

**Figure 1:**
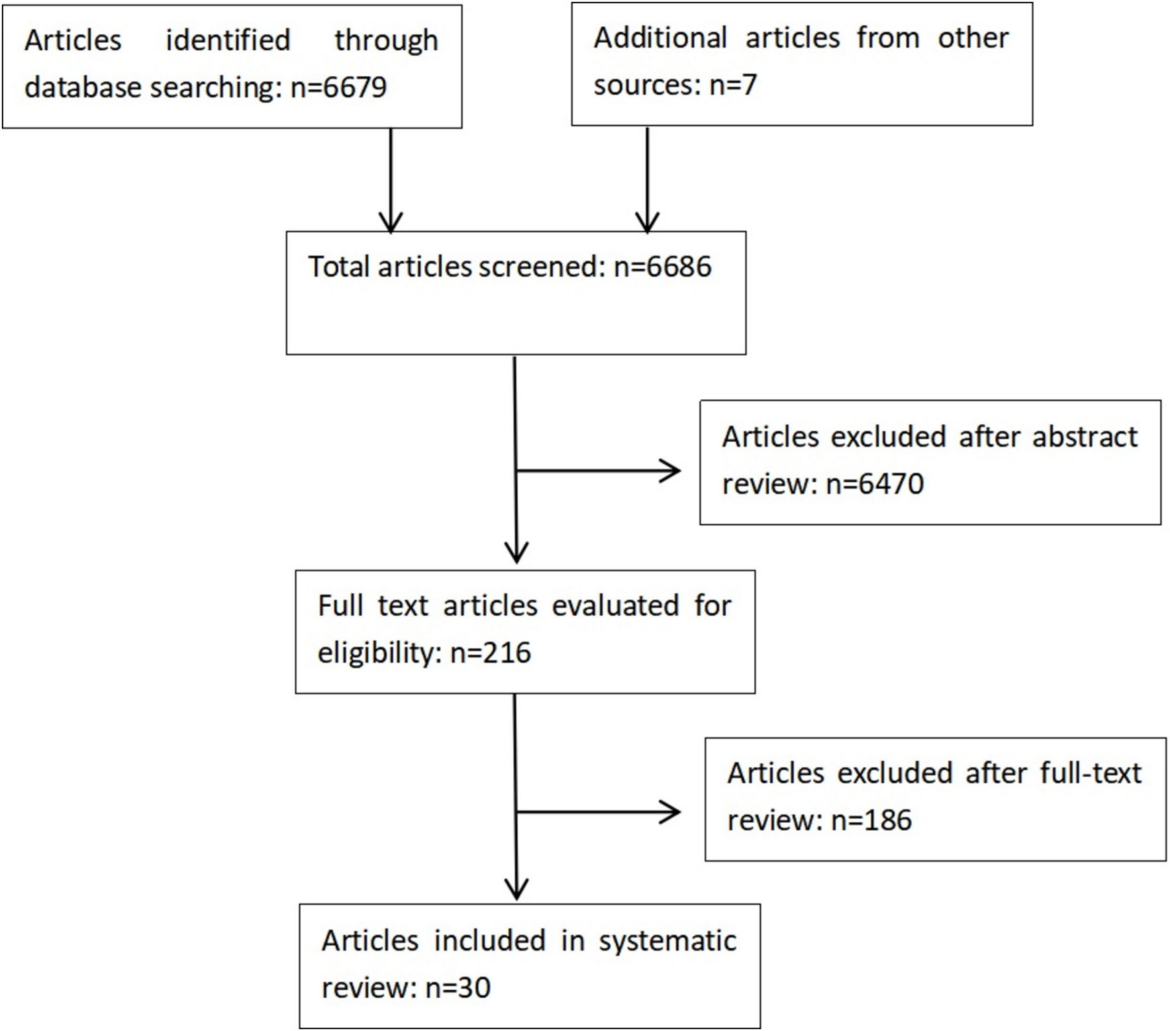
Study selection flowchart of the review.

A total of 6,686 articles were screened, of which 6,470 were excluded following abstract review. After reading the remaining 216 articles in full, a further 186 were excluded. A total of 30 original articles were included in this systematic review (Figure 1). Details of findings are summarized in Tables 1–3 according to training mode.

**Table 1:** The list of the current 3D model for ophthalmic education.

Study	Training objective/device	Trainees	Assessment	Advantage	Disadvantage
Han, R. et al. (2)	Fractures and disorders associated with the orbit area /Three-dimensional Visualization Educational Modeling	First- and second-year ophthalmology residents	Satisfaction was reported 100% by residents	Visualization and mental stabilization; A clear understanding of bones; Spatial visualization with real dimensions; Bones position; Tangible models; Sustainability and a better understanding of content; Better training and easier to learn and memorize	Lack of staining of orbital bones; Lack of separation of skull bones; Uncertain suture of bones; Inadequate models of all disorders
Ramesh, V. et al. (3)	The real-time gonioscopic images and the 110-degree mosaic real-time true color confocal fundus images of various normal and pathological eyes /Eye MG 3D Application	Residents and students	–	Real-time images; providing various multimodal real-time images such as autofluorescence and infrared imaging	–
Halah, H. et al. (4)	Surgical training of ophthalmology / Three-Dimensional Heads-Up Display(HUD)	One hundred and forty ophthalmic surgeons participated in this study. Two-thirds of the participants had 10 years or less experience in the ophthalmology field	Forty-nine (68.1%) of the users found it a valuable educational tool. 60 (83.3%) users recommended its use in surgical training, and 50 (69.4%) recommended using it in general	The peripheral acuity and the depth perception is better in the HUD system than conventional microscopy.	–
Mustafa, S. et al. (5)	To teaching ocular ultrasound /a novel 3D ocular model	Ophthalmology students	–	Providing interactive teaching	–
Xiong, L. et al. (6)	To understand every extraocular muscles’ movement /3D electric ophthalmotrope	Medical students, who were registered for taking ophthalmology course from Medical Collage	By using 5-point Likert scale, The expert’s evaluation score for the imitation effect of medial rectus, lateral rectus, superior rectus, inferior rectus muscle, superior oblique and inferior oblique muscle were 5,5,5,5,3 and 4.The scores of the students with the 3D electric ophthalmotrope model were greater than those with the traditional anatomical model in the in-class quiz and final quiz	Intuitive and vivid	Promotion and application need higher cost and longer time
Famery, N. et al. (7)	Descemet’s membrane endothelial keratoplasty training / Artificial chamber and 3D ed. iris	Beginners and experienced DMEK surgeons	DMEK surgeons were significantly faster than beginners with both graft preparation and insertion time. The performance score correlated with the surgeon’s experience	Close to reality, feasible, resource-sparing and valid	Not all the steps of the Descemet’s membrane endothelial keratoplasty can be permitted to be performed in the model

**Table 2:** The list of the current AI model for ophthalmic education.

Study	Training objective	Trainees	Assessment	Advantage	Disadvantage
Muntean, A. et al. (8)	Personalized Ophthalmology Residency Training	Ophthalmology Residents	The OSCE examination was used for the standardized evaluation of clinical performance of the Ophthalmology Residents	A daily case (real or from the Resident dataset) to solve; Personalized cases based on their needs with better coverage of the disease spectrum; Cases with a level of difficulty adapted to their current performance; A more uniform distribution of similar cases during the 1 year of practice; Feedback from the expert physician	–
Fang, Z. et al. (9)	Artificial intelligence-based pathologic myopia(PM) identification system in the ophthalmology residency training	Ophthalmology residents	All participants were satisfied and agreed that the AI-based PM identification system was effective and helpful to acquire PM identification, myopic maculopathy (MM) classification, and “Plus” lesion localization	Efficiency, convenience, innovative design, flexible learning style, self-learning ability, and self-motivation	Concerns about the hardware requirement, accuracy, and website stability of the AI-based platform
Han, R. et al. (10)	Diabetic retinopathy grading (DR) training	Junior ophthalmology residents and medical students	Through training, the average kappa score (calculated by combining the harmonic means of the DR classifications) was elevated	A feasible reading training method to improve the DR reading level of junior ophthalmologists	–

**Table 3:** The list of the current VR model for ophthalmic education.

Study	Training objective/device	Trainees	Assessment	Advantage	Disadvantage
Kekunnaya, R. et al. (11)	Virtual bedside clinics in pediatric ophthalmology and strabismus /the Zoom platform	Ophthalmology residents, fellows, sub-specialty ophthalmologists, general ophthalmologists, and optometrists	Comparing its effectiveness with conventional bedside clinics, respondents suggested that virtual bedside clinics were better/equally effective in the following techniques: general examination (96%), ocular motility (93.3%), nystagmus evaluation (93.3%), and anterior (80%) and posterior segment examination (73.3%)	Most of the bedside manners, procedural skills, and examination techniques can be effectively taught through this virtual platform with a scope to improve anterior and posterior segment examination skills	5% of the respondents felt that the duration of the virtual individual session was too long/too short
Frisbie, J. et al. (12)	Virtual Medical Student Clinical Rotation for Ophthalmology /the Zoom platform	Medical Students	–	This technology can be utilized in the future not only to help increase exposure to the field, but help limit costs associated with traveling for rotations and create time-efficient opportunities for students wishing to gain ophthalmologic clinical experiences	–
Ramesh, V. et al. (13)	Virtual ophthalmic webinars / Green Mat technology	Ophthalmic postgraduate residents	–	Good clarity, interactivity, and creativity	–
Chan, P. et al. (14)	Diagnostic competency in retinopathy of prematurity (ROP) / tele-education system	Ophthalmology residents	Statistically significant changes of sensitivity for the diagnosis of plus disease, zone, stage, category, and aggressive posterior ROP and specificity for identification of stage 2 or worse were observed (*p* < 0.05)	Easy to use; feedback provided at the end of each case; more effectively to learn	–
Succar, T. et al. (15)	Virtual Ophthalmology Clinic learning / The Virtual Ophthalmology Clinic	Medical students	56% of students rated it more effective than traditional methods	Helpful in medical students’ understanding of how an ocular history relates to the site of pathology	–
He, B. et al. (16)	Ophthalmology learning /Medskl.com	Medical students	Students found the greatest utility of modules was scheduling flexibility (92%) and individualization to learning needs (77%)	Inequities in accessing high-quality ophthalmology education can be mitigated with virtual learning	–
Saleh, M. et al. (17)	Cataract surgery training /Eyesi virtual reality (VR) simulator	Ophthalmic trainees with 2 h or less of simulation and intraocular surgical experience	Trainees’ overall performance improved significantly in the second attempt compared to the first time. (*p* < 0.0001)	Offering the quantitative description of trainees’ performance	–
Nilesh, R. et al. (18)	Continuous curvilinear capsulorhexis training /the Kitaro DryLab model (Frontier Vision Co., Ltd.), SimulEYE SimuloRhexis model (InsEYEt, LLC), and the Bioniko Rhexis (Bioniko Consulting LLC)	Ophthalmic surgeons	For the realistic simulation experience, the Kitaro, SimulEYE models and Bioniko model were 4.56 ± 0.84, 4.19 ± 0.92 and 1.38 ± 0.80 on a 7-point modified Likert scale	Providing high realistic simulation experience	–
Meredith, Weiss. et al. (19)	Endoscopic endonasal dacryocystorhinostomy training / the endoscopic endonasal surgery simulator	Ophthalmology residents	Residents who trained on the simulator performed significantly better compared with the group who trained on cadaver	Providing a quantified measure of a novice surgeon’s preparedness to perform surgery; as a teaching tool, the simulator offers the potential for standardization in resident education	–
Elisabeth, Feudner. et al. (20)	Capsulorhexis / the Eyesi (VRmagic, Mannheim, Germany) ophthalmic virtual reality surgical simulator	31 medical students and 32 ophthalmological residents in postgraduate year (PGY) 1 to 5	Residents also showed marked improvement for time scores whereas no obvious difference in time scores was observed for students. For students and residents taken together, subjects with a low number of attempts on training had a higher probability to perform well than the group with a higher number of attempts	The potential exists to train a surgical novice to a high level of objectively measured skill before he or she is permitted to operate on a patient, and thus to improve the safety of patients facing surgery by a surgical novice.	No haptic feedback and fluid dynamics; Corneal or scleral incisions or suturing techniques cannot be practized
Cécile, C. et al. (21)	Vitreoretinal modules / the Eyesi (VRmagic, Mannheim, Germany) ophthalmic virtual reality surgical simulator	Fifteen residents with no vitreoretinal experience and six trained vitreoretinal surgeons (>100 procedures per year)	Experienced vitreoretinal surgeons outperformed residents with regard to the overall score on the navigation 1 (*p* = 0.01), forceps 1 (*p* < 0.01), epiretinal membrane peeling modules 1 and 2 (*p* = 0.02) and ERM2 (*p* = 0.04) modules	–	–
Charlotte, J. et al. (22)	Vitreoretinal surgical skills / the Eyesi (VRmagic, Mannheim, Germany) ophthalmic virtual reality surgical simulator	10 junior residents without any surgical experience, 8 senior residents with prior experience in cataract surgery and 5 vitreoretinal surgeons	Senior residents significantly improved their simulator skills over time, reaching a plateau at the fifth iteration and equaling expert performance (*p* = 0.420)	–	–
Sukanya, M. et al. (23)	Vitreoretinal surgical Training / the Eyesi (VRmagic, Mannheim, Germany) ophthalmic virtual reality surgical simulator	Retina fellows-in-training	Most (*n* = 25, 68%) respondents considered surgical simulators to be the best training tool before operating on the human eye. The majority (*n* = 33, 89%) of participants responded that VR surgical skills acquired during simulator training were transferrable to the operating room	–	–
Patrick, S. et al. (24)	Learning cataract surgery / the Eyesi ophthalmic virtual reality surgical simulator	The second year of ophthalmology residency for novice postgraduate year 3 (PGY-3) residents	The addition of surgical simulation training was associated with a significantly reduced rate of complications	–	–
Colin, M. et al. (25)	Continuous, curvilinear capsulorhexes (CCCs) during cataract surgery / the Eyesi (VRmagic, Mannheim, Germany) ophthalmic virtual reality surgical simulator	Resident surgeons at a teaching hospital with level of postgraduate year (PGY)	There was a statistical trend toward fewer errant CCCs among PGY 4 (14.6%) compared with PGY 3 (22.8%) surgeons (*p* = 0.12)	–	–
Colin, M. et al. (26)	Cataract surgery training / the Eyesi ophthalmic virtual reality surgical simulator	Resident postgraduate year (PGY)	Errant CCC was occurred in 24 of 47 (51.1%) in the “No Eyesi” group, and 8 of 58 (13.8%) in the “Capsulorhexis Intensive Training Curriculum for the Eyesi” group (*p* = 0.00016)	–	–
Tran, L. et al. (27)	A practice trial in the anterior segment training module, followed by 3 scored trials in the anterior forceps, antitremor, and capsulorhexis modules / the Eyesi (VRmagic, Mannheim, Germany) ophthalmic virtual reality surgical simulator	4 medical students, 4 ophthalmic medical technologist trainees, 36 ophthalmology residents, 3 fellows, and 18 staff ophthalmologists	Participants with greater experience achieved significantly higher total scores than those who were less experienced(*p* = 0.011), with lower total task time (*p* = 0.044) and fewer injuries to the cornea (*p* = 0.001) and lens (*p* = 0.026)	–	Costly
Shameema, S. et al. (28)	Capsulorhexis Training of cataract surgery / MicroVisTouch™ (ImmersiveTouch, Chicago, IL, United States)	78 ophthalmology residents	The improvement in all test variables was statistically significant (*p* < 0.05)	–	–
Chee, L. et al. (29)	Four main phacoemulsification cataract surgery procedures: corneal incision, capsulorhexis, phacoemulsification and intraocular lens implantation (IOL)/ prototype	10 experienced ophthalmologists and 6 medical residents	Subjects with greater experience obtained significantly higher scores in all four main procedures. Positive correlation was observed between experience and anti-rupture	the VR simulators provide training modules for all the four main procedures of phacoemulsification cataract surgery	–
Ayşe, O. et al. (30)	Cataract surgical training / Eyesi simulator	Ophthalmic residents	–	Ideal to evaluate the effect of surgical environment on surgeon performance, including tiredness, visual acuity, use of the nondominant hand	–
Ya, H. et al. (31)	Chopping in cataract surgery/ Eyesi simulator	Ophthalmology residents	The residents in surgical-simulator training group got less corner area injured, and they spend less time than wet-lab training group (*p* < 0.05)	–	In wet lab, if the incision stress increased, the cornea would be deformed, which may blurred the clarity of visual field under surgical microscopy. The residents would notice this phenomenon. However, the residents in the by simulator might ignore it

## 4 Novel techniques in ophthalmology education

### 4.1 Three-dimensional technology

In recent years, three-dimension (3D) technology has developed extensively in medical education, and it brings many advantages. 3D technology allows for the creation of detailed, interactive anatomical models that provide a more realistic representation of the human body compared with traditional 2 dimensional images or diagrams. This enhanced visualization can help students to gain a better understanding of anatomical structures and spatial relationships. Interactive 3D models and simulations can increase student engagement and motivation by providing a hands-on learning experience. 3D technology can be used to create realistic medical simulations that replicate clinical scenarios and procedures, enabling students to practice and refine their skills in a safe and controlled environment before working with real patients, which improves their confidence and competence. 3D technology can also be tailored to individual learning needs, allowing students to study at their own pace and focus on areas where they need additional practice. 3D technology has been used in ophthalmic education, and advantages and disadvantages are summarized in Table 1.

### 4.2 Better depth perception and closer to reality

3D models can provide a clear understanding of complicated spatial structures. A study by Vatankhah et al. (2) focused on the use of 3D models derived from CT scans for ophthalmology education. The researchers designed 3D models to aid in the visualization of complex anatomical structures, particularly in the orbital area. Satisfaction was reported as 100% by study participants. The results showed that the models significantly improved the participants’ understanding and retention of anatomical knowledge, highlighting the potential of 3D models as valuable educational tools. Ramesh et al. (3) described the development and application of the “Eye MG 3D” app. This app has a comprehensive 3D atlas of ocular anatomy and pathophysiology with real-time TrueColor confocal images, providing better understanding for students of complicated ocular anatomy and pathophysiology.

The superior benefits of peripheral acuity and depth perception in 3D surgical training are evident in current literature. Bin Helayel et al. (4) investigated the benefits of using a 3D heads-up display system compared with conventional microscopy for ophthalmic surgeries. Most participants (83.3%) recommended its use in surgical training due to its superior educational value. Mustafa et al. (5) presented a 3D educational tool designed to enhance the teaching of ocular ultrasound, which could improve trainees’ spatial awareness and understanding of ocular structures.

These educational models are intuitive and vivid, close to reality, and are able to provide better understanding and visualization. Lei et al. (6) developed a novel 3D electric ophthalmotrope to enhance the teaching effectiveness of ocular movements in ophthalmology. In the study, seven experts evaluated the 3D electric ophthalmotrope’s simulation ability and precision. Compared with the traditional anatomical model, the experts agreed that the 3D electric ophthalmotrope was easier for students to understand every extraocular muscle’s movement in each evaluation index. A randomized controlled trial demonstrated superior performance in both in-class quizzes and final exams for students taught with the 3D model compared with those using traditional models.

### 4.3 Feasible and resource-sparing

Famery et al. (7) presented a model for teaching Descemet membrane endothelial keratoplasty (DMEK) on an artificial anterior chamber with a 3D-printed iris. The study concluded that this model provided a resource-efficient opportunity for trainees.

### 4.4 Unable to simulate all the structures and all the steps of surgical procedures

In Vatankhah’s study (2), for example, the 3D printed skull lacked separation of the skull bones and was also unable to provide simulations for all orbital disorders. A study by Famery et al. (7) used a 3D-printed model to provide training for DMEK; however, the descemetorhexis part of the surgery could not be performed with the model.

### 4.5 Artificial intelligence for ophthalmology education

There are numerous advantages to using artificial intelligence (AI) in medical education. AI algorithms can tailor educational content to the specific needs of each student. AI-powered adaptive learning platforms can adjust the difficulty level of educational materials based on the student’s progress and performance, and AI-powered systems can provide real-time feedback to students on their performance, highlight areas for improvement, and offer personalized recommendations for further study. At present, there have been many studies on the application of AI in ophthalmology teaching, and the results have demonstrated the role of AI as a new technology in the field of ophthalmology teaching (Table 2).

### 4.6 Personalized training

Muntean et al. (8) presented a study investigating the use of AI for personalized ophthalmology residency training. The study focused on the development of an AI-based framework to enhance residency programs by ensuring a balanced distribution of cases among residents. The AI system matched cases to residents based on their training history and performance, with feedback from attending physicians to continually update residents’ portfolios. This approach could improve precision medical education by personalizing learning experiences and standardizing the training process for ophthalmology residents.

### 4.7 Promotion of self-motivation

A study by Fang et al. (9) evaluated the effectiveness of an AI-based pathologic myopia (PM) identification system in an ophthalmology residency training program. Results showed significant improvement in post-lecture scores for the group using the AI-based PM identification system compared with the group trained by traditional lectures. Evaluations by the residents revealed that the AI training model was perceived as better motivation than traditional didactic lectures.

### 4.8 Providing efficient, convenient, and flexible learning

AI training models also provide an efficient, convenient, and flexible learning style. A study by Fang (9) revealed that residents perceived the AI-based system to be effective, efficient, and beneficial for understanding PM identification and classification. Han et al. (10) investigated the efficiency of an AI reading label system for diabetic retinopathy grading training among junior ophthalmology residents and medical students. The AI system was found to be an effective training tool, significantly improving the diagnostic accuracy of junior ophthalmologists.

### 4.9 High demand for hardware devices

AI training models have many advantages. However, as mentioned by Fang (9), these advanced educational models need qualified hardware and a stable website for the AI-based platform, which affects their wide application.

### 4.10 Virtual education for ophthalmology education

There are significant advantages to virtual reality (VR) technology in medical education. VR simulations can replicate a wide range of medical scenarios, from surgical procedures to patient consultations, providing students with valuable hands-on experience in a safe and controlled environment. This enables students to practice skills and decision-making under realistic conditions, which improves their confidence and competence. Many VR technologies have already been applied to ophthalmology education (Table 3).

### 4.11 Stimulation of clinical procedures

Procedural skills and examination techniques can be effectively taught through a virtual platform to improve anterior and posterior segment examination skills. Kekunnaya et al. (11) piloted an innovative teaching method through live virtual bedside clinics for pediatric ophthalmology and strabismus, comparing its effectiveness with conventional bedside clinics. Over 95% of 287 survey respondents found that the virtual clinics were equally or more effective than traditional methods in teaching physical examination, clinical knowledge, reasoning, procedural skills, and communication.

### 4.12 Cost-effective

Frisbie et al. (12) developed a novel virtual clinical rotation for ophthalmology medical students at the University of Maryland School of Medicine, integrating mobile-mounted tablets, which allowed students to participate in inpatient consults, clinics, and ophthalmic surgeries remotely. The program included independent learning modules, video lectures, interactive sessions, and virtual wet laboratories. Feedback indicated high effectiveness in simulating in-person experiences, increased interactions with residents and faculty, and reduced costs associated with traveling for rotations. Ramesh et al. (13) introduced an innovative “green mat” technology—this cost-effective approach has proven beneficial for continuing medical education.

### 4.13 Remote learning

Chan et al. (14) described a tele-education system developed to improve diagnostic competency in retinopathy of prematurity (ROP). The tele-education system for ROP education was found to be easy to use and effective in improving the diagnostic accuracy of ophthalmology residents. This system may be useful in both healthcare and medical education reform settings by creating a validated method to certify telemedicine providers and educate the next generation of ophthalmologists.

### 4.14 Effective educational tool

Succar et al. (15) assessed the impact of the Virtual Ophthalmology Clinic (VOC) on medical students’ learning with a randomized controlled trial involving 188 students from the University of Sydney. Participants were divided into experimental and control groups, with the former using the VOC and the latter receiving traditional hospital-based teaching. Results revealed a significant improvement in the experimental group’s knowledge and long-term retention of ophthalmic information. At 12 months follow-up testing, the experimental group scored, on average, higher than the controls. The VOC was highly rated for its ability to enhance clinical reasoning and history-taking skills, demonstrating its effectiveness as an educational tool.

### 4.15 Providing equal educational opportunities

He et al. (16) conducted a survey to evaluate the effectiveness of a virtual ophthalmology education program among Canadian medical students. The program—which included synchronous webinars and asynchronous video modules—was well-received by students, who reported increased access to ophthalmology education and reduced feelings of social isolation. The authors suggested that inequities in accessing high-quality ophthalmology education can be mitigated with virtual learning.

### 4.16 Standardization of education

A VR study model can also offer a quantitative description of trainees’ performance. Saleh et al. (17) evaluated the variability of performance among novice ophthalmic trainees using the EyeSi VR simulator. The study quantified the reproducibility of novice performance with the VR tool. Studies have shown that VR surgical simulators can provide a highly realistic simulation experience (18). As a teaching tool, the simulator has potential for standardization in resident’s education (19). VR training can provide quantitative evaluation for trainees under the same training standard and allow as much training as necessary to achieve the training goal without the need for continuous supervision or taking surgical risks (20–31).

### 4.17 Inability to simulate all the steps of surgical procedures

VR simulators were unable to provide all the surgical training steps. The EyeSi’s—the most widely applied ophthalmic simulator worldwide—cataract training modules, for example, do not include corneal incision and intraocular lens implantation, which are essential steps in cataract surgery training (20–27, 29–31).

### 4.18 Inability to simulate all the situations of surgical procedures

Existing ophthalmic simulators cannot simulate all the situations in real surgery. In the wet lab, if incisional stress increased, the cornea would be deformed, which may blur the clarity of the visual field under surgical microscopy,—ophthalmology residents would notice this phenomenon. However, residents using a simulator have been found to ignore it (31). Most ophthalmic simulators have no haptic feedback. As discussed by Feudner et al. (20), on EyeSi, the only haptic input comes from the fulcrum effect of the rigid plastic wall of the eye model; there is no haptic feedback from intraocular tissues. In contrast, during real surgery, there is haptic feedback from intraocular tissues as well as from the eye wall. Moreover, corneal and scleral rigidity are not constant but may change considerably during surgery depending on intraocular pressure (20). Ophthalmic simulators have no fluid dynamics. Feudner et al. (20) also noted a drawback with regard to fluid dynamics—to date, changes of anterior chamber depth (e.g., due to fluid leakage because of excessive incision stress) are not simulated; however, they are an important source of surgical complications.

### 4.19 Costly

As mentioned by Tran et al. (27), ophthalmic simulators are costly and cannot be used in all institutions.

## 5 Discussion

Vision has a significant impact on the quality of life. Misdiagnosis and delayed diagnosis lead to demonstrated poor outcomes, including permanent vision loss and severe pain (1). High-quality ophthalmic education is of vital importance to assist patients to maintain ocular health. Effective education could help junior ophthalmologists to identify and manage various eye conditions promptly, thereby preventing further damage and preserving vision.

A recently published systematic (1) review demonstrated that the amount of ophthalmology teaching in medical schools has been declining globally for two decades. In ophthalmology assessments, on average, students do not score highly for either their knowledge or their skills. Approximately three-quarters of students are not confident in their knowledge, and two-thirds are not confident in their skills. Spencer et al. (1) noted that most studies note that a reduction in the length of ophthalmology courses over the last 20 years is the reason for the decline in ophthalmology education globally. A nationwide survey of 840 medical students and junior doctors in Australia found that only five participants believed that the ophthalmology teaching they had received was sufficient (32).

Ophthalmology educational technologies continue to evolve, which may help to bridge the gap in declining teaching time and may result in a trend away from didactic lecture-style education to methods with improved student interaction, enjoyment, and time efficiency.

Additionally, the traditional teaching method of ophthalmology is deficient. Surgical education has traditionally relied on an apprenticeship teaching model. The quality of teaching is highly dependent on the mentor’s surgical and teaching abilities, which leads to variability in how trainees learn and acquire surgical skills (33). While various assessment tools have been created to standardize the evaluation of surgical skills, these modalities are subjective because the grading is performed by humans (34, 35). Moreover, the use of these tools remains limited in clinical practice and surgical curricula, partially due to resource limitations, which require an expert observer for grading (36, 37). Quantifiable, objective, and standardized methods to teach and assess surgical skills, especially between mentors and across institutions, are currently lacking in surgical education.

The application of 3D technology is noteworthy as a useful tool in surgical training. The application of 3D simulators in medical training has been transformative, offering numerous benefits and opportunities to improve medical education and patient outcomes. 3D simulators can replicate the exact anatomical structures of patients and provide highly realistic models for training, which are essential for understanding spatial relationships and practicing surgical techniques. Practicing on a 3D simulator could reduce the risks associated with the first-time performance of new or intricate surgical procedures on actual patients. Compared with traditional surgical training methods, such as cadaveric dissection or live animal models, which can be expensive, 3D simulators offer a more cost-effective and accessible alternative (38). 3D models have been used increasingly for medical education, with research highlighting their value in different types of surgery, including oral and maxillary (39–41), otorhinolaryngology (42–50), neurosurgery training (51–56), orthopedics (57, 58), and urinary surgery training (59–62).

The numerous advantages of 3D teaching models are also present in ophthalmology education. The 3D model of orbit (2), the extraocular muscles model (6), and the artificial chamber (7) have demonstrated that 3D models are intuitive and vivid education tools, with real spatial visualization, thereby providing a highly realistic simulation experience. These 3D models also have been identified as feasible and valid training tools, which are easy to use and could provide convenient and flexible learning for medical students (2, 7).

AI technology—a constantly developing field—has made significant progress in education, health, industry, and many other sectors. These technologies, especially in education and learning, have great potential to provide students with personalized learning experiences, optimize learning processes, provide additional resources, and improve the quality of education (63). AI models in ophthalmic education are feasible training tools, which can provide personalized training (8), promote self-motivation (9), and create time-efficient opportunities for students (12).

Clinical teaching is often conducted using real patients, and this method can sometimes be very challenging due to the comfort of patients or time constraints in a busy clinic. Due to the increased health consciousness and awareness of the public, patients often expect the doctors to be experienced instead of being treated as “guinea pigs” by students who have just entered residency training or are still in medical school (64).

Kekunnaya’s study (11), which compared the effectiveness of conventional bedside clinics with that of virtual clinics, found that virtual bedside clinics were better/equally effective for the following techniques: general examination (96%), ocular motility (93.3%), nystagmus evaluation (93.3%), and anterior (80%) and posterior segment examination (73.3%). Most bedside manners, procedural skills, and examination techniques can be effectively taught through this virtual platform, with potential to improve anterior and posterior segment examination skills. This also helps residents to build confidence without causing any harm to the patient.

VR training models can train surgical novices to a high level of objectively measured skill before they are permitted to operate on a patient, thus improving patient safety when facing surgery by a surgical novice (20–31). Most models can provide a quantitative description of the trainee’s performance. As a teaching tool, the models have potential for standardization in residents’ education (20–27, 30, 31).

Advanced educational techniques share similar advantages, for example, they are all intuitive and vivid training tools (2, 6, 7, 18), and they are all effective, flexible, and convenient to use (2, 7, 9–11, 14). However, these high-tech modern educational techniques also have common defects. Neither 3D training models nor VR educational models can simulate all the procedures of ophthalmic surgeries (7, 20–27, 30, 31). More importantly, all these models are unable to simulate the complete real experience of clinical practice. To use cataract surgery training as an example, incompetent control of fluid dynamics could lead to changes of anterior chamber depth, which is a significant cause of cataract surgical complications. None of the training models can currently provide simulation of fluid dynamics (17, 18, 20, 24–26, 28–31). Therefore, in spite of numerous advantages, conventional education, especially in surgical training, cannot be replaced.

Nevertheless, the development and promotion of advanced ophthalmic educational techniques have enormous significance. These are effective training tools that can promote the clinical ability of students (4, 6, 9–11, 14, 15, 17, 19, 20, 22–26, 29, 31). They are easy, convenient, and flexible to use (8–10, 13) and can create more time-efficient opportunities for students. They are also resource-sparing (6, 11). Such tools can promote students’ self-learning ability and self-motivation (8). They can also mitigate the inequities in accessing high-quality ophthalmology education (15).

## 6 Conclusion

The application of 3D technology, AI, and virtual education methods in ophthalmology education is significantly advancing. These approaches not only improve knowledge retention and practical skills but also make education more accessible and engaging. As these technologies continue to evolve, their integration into medical curricula holds promise for further enhancing ophthalmology training and practice.
